# Progressive Hemifacial Atrophy and Linear Scleroderma En Coup de Sabre: A Spectrum of the Same Disease?

**DOI:** 10.3389/fmed.2017.00258

**Published:** 2018-01-31

**Authors:** Irina Khamaganova

**Affiliations:** ^1^Pirogov Russian National Research Medical University, Moscow, Russia

**Keywords:** progressive hemifacial atrophy, linear scleroderma en coup de sabre, clinical features, histopathological findings, predisposing factors

## Abstract

Similar clinical and histhopathological features in progressive hemifacial atrophy and linear scleroderma en coup de sabre are well known. Trauma may predispose to the development of both diseases. The lack of association with anti-Borrelia antibodies was shown in both cases as well. The otolaryngological and endocrine disorders may be associated findings in both diseases. However, there are certain differences in neurological and ophthalmological changes in the diseases.

## Introduction

The Parry–Romberg syndrome has an old history. Appenzeller and colleagues scrutinized about 200 mummy colored portraits painted at the beginning of the first millennium. The Parry–Romberg syndrome was diagnosed in two persons (1).

Charlier and colleagues retrospectively described the disease in a major French revolution leader Mirabeau (2).

The “Collections from the Unpublished Medical Writings of the Late Caleb Hillier Parry” (1825) presented the first description of Parry–Romberg syndrome (3). Romberg described the disorder in 1846 (4). Eulenburg proposed the title “progressive hemifacial atrophy” (PHA) in 1871 (5).

Tolkachjov and colleagues (6) named the synonyms of Parry–Romberg syndrome: idiopathic hemifacial atrophy, PHA, and Romberg syndrome. Otherwise, Nomura and colleagues pointed out that PHA could progress in lupus profundus, lipodystrophy, morphea, and Parry–Romberg syndrome (7). The term PHA is often used as a synonym of Parry–Romberg syndrome.

In spite of the long history, the Parry–Romberg syndrome has unknown etiology and pathogenesis. Earlier it was highlighted that PHA and linear scleroderma “en coup de sabre” (ECDS) often coexisted (6, 8), but some authors described PHA as a unique disease (9–11).

The problem of differential diagnosis of PHA and ECDS is being discussed (12, 13). The goal of the review is to analyze the data on these opinions.

## Material

The original articles, reviews, and cases from Medline PubMed were the main source of information. The words “progressive hemifacial atrophy,” “Parry–Romberg syndrome,” “localized scleroderma en coup de sabre,” “linear scleroderma en coup de sabre,” and “linear morphoea” were used for the search. The inclusion criteria contained:

– publication no later than 2001.
– publication presenting the problems named in the search words.

The exclusion criteria contained:

– publications later than 2001.
– articles on another subject 5,479 papers published from March 1897 to August 2017 were analyzed.

We excluded 5,100 papers published before 2001 as far as they were earlier discussed in some reviews (6, 8, 10). We selected 125 publications that presented clinical and histopathological characteristics of PHA and ECDS, predisposing and provocative factors for their progressing. 254 articles that were written on other topics were excluded.

## Results

### Clinical and Histopathological Features of PHA and ECDS

Our results are provided in Table 1. So, Tolkachjov and colleagues (6) summarized the published data on the relationship of ECDS and PHA. The average age of the beginning in ECDS was 10 years, in PHA 13.6, respectively. Both diseases have female predilection (6, 14, 15) and started in childhood previously (16).

**Table 1:** Clinical and histopathological findings in progressive hemifacial atrophy (PHA) and en coup de saber (ECDS) (1).

Clinical findings	PHA	ECDS
Average age and gender	13.6 years (6) Female predilection (6, 14, 15)	10 years (6) Female predilection (6, 14, 15)

Abnormalities of skin and mucosa	The involvement of half of face prevails, the lesions are presented by paramedian atrophy, absence of underlying skin induration, atrophy of subcutaneous tissue, fat, muscle atrophy, and deformity of tongue, teeth, and gingival (6, 17), in some cases accompanied by orthodontic and maxillofacial changes (18–20)	The predominant localization is on scalp to forehead, the lesions progress with a proliferative and inflammatory phase and later atrophy and residual deformity, the scalp involvement is usually accompanied by hair loss (6, 21–27), oral involvement is unusual (28)

Histopathological findings	Subcutaneous fat atrophy, decrease of adnexal structures, and mononuclear cell infiltrates (6)	Sclerosis of the skin and underlying tissues due to excessive collagen deposition, adnexal atrophy, and mononuclear cell infiltrates, perineural inflammation is common (29, 30)

Neurological pathology	Trigeminal neuralgia, facial paresthesia, severe headache, and epilepsy are the most common complications (31–33)	Seizures, focal neurological deficits, headache, and neuropsychiatric changes are not rare (34, 35)

Ophthalmological disorders	The corneal and retinal changes were named as the most common ocular disorders, the most frequent periocular changes were enophthalmos, eyelid, and orbit alterations (36)	Ophthalmological symptoms are rare (37)

Otorhinolaryngological disorders	Otorhinolaryngological disorders are rare (38, 39)	Otorhinolaryngological disorders are rare (27)

Endocrine disorders	Endocrine disorders may be associated findings (28, 40, 41)	The occurrence of ECDS was described in a woman in the 33rd week of pregnancy (42)

Viral and bacterial infections	The disease can be provoked by viral infections (43), the lack of association with anti-Borrelia antibodies was shown (44)	The lack of association with anti-Borrelia antibodies was shown (44)

Trauma	Trauma may predispose to the development of the disease (45–49)	Trauma may predispose to the development of the disease (50)

The PHA usually presents in the first 20 years of life, late onset is rare (6). The disorder is usually slowly progressive. The defect becomes more pronounced in long duration, leading to esthetic and functional deficits (51). Patients may experience halting of the facial atrophy. But in fact, it may be difficult to differentiate disease acceleration from normal evolution. The PHA main clinical features included paramedian atrophy, absence of underlying skin induration and atrophy which might extend down entire face (6). Typical cases demonstrate the involvement of one or more branches of the fifth cranial nerve. The atrophy of such structures as subcutaneous tissue, fat, muscle and osteocartilaginous was revealed in PHA patients. The changes create a sunken hemiface appearance (6, 17). As far as it is “hemifacial” the involvement of half of face is usual, but Pathi and colleagues reported a case of involving one-half of the body (52).

En coup de sabre is usually a linear unilateral depression on the frontoparietal scalp or paramedian forehead. ECDS develops through the stages of proliferation, inflammation phase, atrophy, and residual deformity. The predominant localization is on scalp to forehead (6, 21–23). The abnormalities of the skin, subcutaneous tissues, and fascia were shown in localized scleroderma (24, 25). Besides, the atrophic shiny plaque may be associated with madarosis (6).

The scalp involvement was usually accompanied by hair loss (26, 27). The lesion really resembles “stroke from a sword” (“en coup de sabre”) (6). Gradual progress over 60 years was described (53). The outcome of the disease is characterized by certain variety from cosmetic problem to invalidism (6, 23, 53).

The atrophy and deformity detected in PHA were noted in the tongue, teeth and gingiva (6). Verma and colleagues reported a PHA patient with marked left-sided facial atrophy and wasting of the tongue (49). Mucosa can be involved in PHA (54) and in ECDS (28). In some cases mucosal involvement may be accompanied by orthodontic and maxillofacial changes (18, 20). ECDS and PHA can be deforming and irreversibly disabling (55, 56). Segna and colleagues emphasized the gravity of the associated deformity and its impact on facial function and appearance (57).

As for scleroderma, xerostomia, microstomia, idiopathic resorption of tooth and mucosal erosions are diagnosed previously in diffuse scleroderma (58). Oral involvement in ECDS is rare, and it may be presented by white linear scar-like fibrotic areas, atrophical tongue papillae, gingival recession, and alveolar bone resorption (28). Hørberg and colleagues presented a case of ECDS in 6-year and 10-month-old Turkish girl. The dental clinical and radiographic examination was performed. The malformed left maxillary incisors with short roots and lack of eruption were revealed (59). A progressive recession on teeth 11 and 12 was described in 19-year-old patient with ECDS (60). A progressive recession on gingiva was shown in 13-year-old girl with ECDS as well (28).

Bilateral involvement seems to be more common in PHA and in ECDS than previously reported (8).

The histopathological examination revealed dermal sclerosis in both diseases. Besides, PHA was characterized by subcutaneous fat atrophy, decrease of adnexal structures, and mononuclear cell infiltrates (6). In ECDS, sclerotic changes of the skin and hypoderma due to excessive collagen deposition, adnexal atrophy, and mononuclear cell infiltrates were revealed as well (6, 61). Walker and colleagues underlined that severe inflammation might lead to pain and functional limitation in morphea (62). Perineural inflammation is common in morphea (29). Goh and colleagues described a substantial perineural lymphocytic and plasmacytic infiltrate, extending into deep layers in ECDS patient (30). Pierre-Louis and colleagues presented ECDS patient with a 1.5-year history of linear morphea and alopecia with morphological changes presented by atrophic follicular remnants (63).

### Neurological Pathology

The extracutaneous changes in PHA were noted as well. The neurological complications are the most frequent (6, 64). Chokar and colleagues presented a PHA case with alien-hand syndrome (65).

Okumura and colleagues (66) performed a detailed neuroimaging study in a PHA patient. Parents noticed asymmetry of the face at 4 months of patient’s age. PHA was diagnosed at 18 months of age. At 22 months of age, patient had widespread white matter lesions in the left hemisphere which presented diminished signal intensities on T1-weighted images and enhanced signal intensities on T2-weighted images and fluid-attenuated inversion recovery (FLAIR) images. Examining of the occipital areas in the right hemisphere showed a similar white matter lesion. No changes were noted while neurologic and electroencephalographic examination. MR angiography was performed at 33 and 44 months. It did not reveal any vascular abnormalities. The single-photon emission (SPECT) was performed at 31 months of age. It demonstrated the blood flow within the white matter in the left hemisphere being 55% of the corresponding region of the right hemisphere. Both conventional MR imaging and diffusion tension imaging showed the widespread white matter abnormalities (66).

So, the absence of pathologic neurologic changes in some cases (67, 68) does not prove the intactness of neural system.

The advanced magnetic resonance imaging (MRI) detected the white matter hypersignal on T2-weighted and FLAIR sequences, leptomeningeal enhancement, intracranial calcifications, and brain atrophy (31).

Vix and colleagues (69) showed that central nervous system involvement was frequent among PHA patients. The seizures, headaches, movement disturbances, neuropsychological changes, and focal symptoms were noted.

The trigeminal neuralgia (32, 33, 70, 71), facial paresthesia, severe headache, and epilepsy are the most common complications of PHA (31–33). Variable neurological pathology was described in PHA patients. So, Verma and colleagues reported mirror movements in a hand of a teenager with PHA and epilepsy (72). Epilepsy (epilepsia partialis continua) can arise from the hemisphere opposite to the side of facial atrophy (73). Asai and colleagues (74) presented a case of PHA syndrome associated with contralateral cerebral atrophy. The facial myokymias were also noted in PHA (75). Seifert and colleagues reported two cases PHA with chronic focal encephalitis (76). Gupta and Patil described PHA with multiple intracranial cysts (77). Yanagishita and colleagues presented a case of dyschromatosis symmetrica hereditaria complicated by intracranial hemangiomas and PHA (78). PHA combined with hemifacial spasm was described (79). Zahlane and colleagues reported a case of PHA associated with complete agenesis of the corpus callosum (80). The abnormalities of brain glucose metabolism in PHA may be observed before anatomical changes and therefore facilitate early diagnosis (71). De Lange and colleagues reported a case of PHA with a giant intracranial aneurysm (81).

Neurological changes were noted in ECDS as well, they are more often described in children than in adults (37, 82). Such neurological manifestations as seizures, focal neurological deficits, headache, and neuropsychiatric changes are not rare in ECDS (34, 35, 83). Recurrent myelitis was described in ECDS (84). Takahashi and colleagues (85) presented an ECDS case in a 39-year-old man. The brain hemorrhage was revealed, but any signs of cerebral vascular abnormalities were not noted. The implication of developmental abnormalities was proposed. Lis-Święty and colleagues examined 20 patients with localized scleroderma affecting the face and head. The symptoms and/or abnormalities in the central nervous system in high-resolution computed tomography and/or MRI were observed in 12 patients (60%). It was concluded that the involvement of central nervous system was not correlated with the clinical course of the facial and head localized scleroderma (86). Brain cavernomas associated with ECDS were described in children (26). Gambichler and colleagues presented an ECDS associated with epileptic seizures, as well oculomotor and facial nerve palsy (87). Holland and colleagues emphasized the underrecognized relationship between neurologic complications and ECDS and highlighted thorough skin examination in patients with unexplained neurologic disease (88). Besides, ECDS can be accompanied by focal neurologic deficits, movement disorders, trigeminal neuralgia, and mimics of hemiplegic migraines (89).

The neurological disorders may precede the appearance of skin lesions (40, 90). Verhelst and colleagues presented hippocampal atrophy and developmental regression as initial clinical feature of ECDS (90).

Bergler-Czop and colleagues presented two cases: a case of a 49-year-old woman with clinical signs of PHA and minor neurological symptoms and a case of a 33-year-old patient with ECDS without any neurological symptoms when only special neurological methods had revealed the presence of CNS tumor (91).

Cranial neuropathies, seizure disorder, and vision loss were diagnosed both in PHA and ECDS (6). The neurological symptoms in both diseases may resemble those in Rasmussen encephalitis (13, 92, 93).

### Ophthalmological and Otorhinolaryngological Disorders

Bucher and colleagues (36) summarized the ocular, periocular, and neuro-ophthalmological findings in PHA The most frequent periocular changes were enophthalmos, eyelid, and orbit alterations. The corneal and retinal changes were named as the most common ocular disorders. The optic nerve, ocular motor, and pupillary dysfunction are the most frequent neuro-ophthalmological disorders (36).

Ayyildiz and colleagues presented a patient with PHA at right facial side who developed granulomatous uveitis and periferic retinal vasculitis in his left eye (94). Fea and colleagues described enophthalmos, uveitis, and iris atrophy in 23-year-old female Caucasian patient with PHA (95). The total atrophy of iris and ciliary body with associated ocular hypotony was describes in PHA patient (8). Kaya and colleagues described severe atrophy and loss of one eye in another patient (96).

According to Careta and Romiti, ECDS is rarely associated with ocular abnormalities, the most part of them noted in children (37). On the other side, a multicenter study showed that ophthalmological symptoms were not unusual in patients with juvenile localized scleroderma, particularly in the ECDS subtype (97). Generally in juvenile localized scleroderma ocular abnormalities include episcleritis, uveitis, xerophthalmia, glaucoma, and papilledema (98).

Otorhinolaryngological disorders are rare in PHA. The association of PHA with dysphonia was described (38, 39).

Otorhinolaryngological disorders are not common in ECDS as well. Pathologic changes may extend into nose rarely (27).

### Predisposing and Triggering Factors

The only one case of familial PHA was described by Anderson and colleagues (99).

The family cases of variable localized scleroderma are presented (100–103), but no cases of ECDS in family.

The involvement of X chromosome monosomy (104), the influence of HLA haplotypes (105), the specific HLA classes are associated with localized scleroderma (106). The overlapping gene expression profiles (107), downregulation of microRNA-196a (108), and role of the CAV1 gene (109) are being examined.

However, the genetic predisposition may be realized due to certain triggers, such as endocrine changes, infections, traumas.

Panda and colleagues presented a case of the development of intermittent unilateral painful spasms of jaw during the fifth month of pregnancy in previously healthy woman. Besides, the patient developed hemifacial and hemitongue atrophy. PHA might be correlated either with some hormonal imbalance or some unknown mechanisms (110).

Unterberger and colleagues reported the neurological disorders such as right sided hemiparesis in association with the occurrence of ECDS and preexisting plaque-morphea in a woman in the 33rd week of pregnancy (42).

Endocrine disorders may be associated findings in PHA (28, 40, 41).

The examination of 17 patients with different types of morphea revealed normal thyroid functions in two patients with linear morphoea (111), but another types of localized scleroderma were accompanied in some cases by thyroid diseases (43, 112).

Progressive hemifacial atrophy can be provoked by viral infections (43). Zhang and colleagues reported a 41-year-old woman with PHA who showed an uncharacteristic “relapsing-remitting” evolution of brain lesions and chronic HBV infection may have triggered the relapse in this case (113).

The role of *Borrelia* infection is being discussed in the development of PHA and ECDS (114, 115). Prinz and colleagues concluded that *Borrelia burgdorferi* might be relevant for the induction of severe morphoea (116). Salpietro and colleagues proposed a possibility of molecular mimicry in PHA patient seropositive for *B. burgdorferi* (117).

The lack of association in PHA and ECDS with anti-Borrelia antibodies was shown (44).

Trauma may predispose to the development of PHA (45–49, 118).

The same is noted in ECDS (50). The development of ECDS was presented by Arif and colleagues in a 26-year-old woman. The lesion located on the frontal and forehead regions. Six years before the examination patient had had a trauma at the same site 6 years back (119). Lipson and colleagues reported a case of congenital ECDS, misdiagnosed since birth as birth trauma. The ECDS was diagnosed in adult (120).

### Coexistence of PHA and ECDS

28–42% of patients have been reported to have coexistence of these diseases. Some findings might overlap in the same patient (6). Blaszczyk and colleagues investigated the relationship of PHA and ECDS by approving the presence and type of central nervous system involvement in both diseases (121). The investigation indicated a close relationship between PHA and ECDS. Dixit and colleagues presented a case of mucosal involvement in morphea, associated with PHA (122). The development or transition of ECDS into PHA in the same physical location was described as well (6, 123).

### Conclusion

The genetic predisposition to ECDS may be realized due to certain triggers, such as endocrine changes, infections, and traumas. The only one case of familial PHA was described. The lack of association both in PHA and ECDS with anti-Borrelia antibodies was shown. Both PHA and ECDS may be correlated with hormonal imbalance. PHA and ECDS have certain similar clinical and histopathological features that may indicate to the same spectrum of diseases. The PHA patients suffer from esthetic and functional deficits which may be seriously complicated by the systemic involvement. The outcome of ECDS may vary from cosmetic problem to invalidism. Some differences can be noted in the diseases belonging to the same group. Mucosal involvement and ophthalmological disorders are more frequent in PHA than in ECDS. Variable neurological disorders were described both in PHA and ECDS. The presented data indicate the need for further neuroimaging studies in PHA and ECDS.

## Author Contributions

The author confirms being the sole contributor of this work and approved it for publication.

## Conflict of Interest Statement

The author declares that the research was conducted in the absence of any commercial or financial relationships that could be construed as a potential conflict of interest. The reviewer AP and the handling Editor declared their shared affiliation.
